# Car drivers coping with hazardous events in real versus simulated situations: Declarative, behavioral and physiological data used to assess drivers’ feeling of presence

**DOI:** 10.1371/journal.pone.0247373

**Published:** 2021-02-19

**Authors:** Elise Gemonet, Clément Bougard, Stéphane Masfrand, Vincent Honnet, Daniel R. Mestre

**Affiliations:** 1 Institut des Sciences du Mouvement, Centre National de la Recherche Scientifique, Aix-Marseille Université, Marseille, France; 2 Groupe PSA, Centre technique de Vélizy, Vélizy-Villacoublay, France; Tongii University, CHINA

## Abstract

More than 1.3 million people lose their lives every year in traffic accidents. Improving road safety requires designing better vehicles and investigating drivers’ abilities more closely. Driving simulators are constantly being used for this purpose, but the question which often arises as to their validity tends to be a barrier to developments in this field. Here we studied the validity of a simulator, defined as how closely users’ behavior under simulated conditions resembles their behavior on the road, based on the concept of drivers’ feeling of presence. For this purpose, the driving behavior, physiological state and declarative data of 41 drivers were tested in the Sherpa2 simulator and in a real vehicle on a track while driving at a constant speed. During each trial, drivers had to cope with an unexpected hazardous event (a one-meter diameter gym ball crossing the road right in front of the vehicle), which occurred twice. During the speed-maintenance task, the simulator showed absolute validity, in terms of the driving and physiological parameters recorded. During the first hazardous event, the physiological parameters showed that the level of arousal (Low Heart Rate/High Heart Rate ratio x10) increased up to the end of the drive. On the other hand, the drivers’ behavioral (braking) responses were 20% more frequent in the simulator than in the real vehicle, and the physiological state parameters showed that stress reactions occurred only in the real vehicle (+5 beats per minute, +2 breaths per minute and the phasic skin conductance increased by 2). In the subjects’ declarative data, several feeling of presence sub-scales were lower under simulated conditions. These results suggest that the validity of motion based simulators for testing drivers coping with hazards needs to be questioned.

## Introduction

Car-driving simulators are an ideal research tool because they can be used to monitor and observe parameters with a high level of reproducibility in a completely safe setting. This makes accident research much more affordable. For instance, rear-end collisions have been estimated to account for 20–30% of all crashes and about 10% of all fatal crashes [1]. In most cases, the driver’s reaction contributes decisively to the outcome of an accident [2,3]. Understanding the causes and consequences of accidents and developing solutions is a major issue for improving road and car safety. It is therefore necessary to ensure that simulators are reliable from the point of view of the human behavior they induce.

### Literature review

#### Presence

Data recorded under simulated driving conditions can only be used if the validity of the simulator has been proved. Participants are expected to respond to events in the same way as they would to normal driving conditions [4]. If the results obtained in a simulator are identical to those recorded in the real world, we speak of absolute validity; if they are found to be similar upon applying a scale factor, we speak of relative validity [5]. From the human factors perspective, the validity of a virtual environment can be measured via the user’s presence. Several subscales have been proposed to facilitate its understanding, such as ethological validity giving an analogy with observed behavior, psychological validity comparing the mechanisms and cognitive cost involved, physiological validity focusing on similarities between physiological reactions, and emotional validity based on differences between emotional states [6,7]. The very few results obtained using open-ended questionnaires of presence after a hazardous event occurs, have shown that motorcyclists tend to forget about the scientific part of the experiment and focus on not falling, which is consistent with how they behave in reality [1,8]. Also, in studies on these lines, presence has often been measured on the basis of behavioral and physiological data, using what has been called the "field truth" paradigm [9,10]. One of the main advantages of this approach is that the parameters of interest can be measured in real time.

#### Driving behavior

Driving behavior is the central component of ethological presence in a simulator. One of the tasks most frequently used in dynamic simulators consists in following a vehicle which brakes suddenly. In this situation, depending on the studies, authors observed a braking reaction only from 15% to 85% cases, braking combined with avoidance from 15% to 85% cases and only avoidance maneuvers from 0% to 15% cases. Venkatraman, Lee & Schwarz [11] studied this aspect more closely and concluded that the nearer drivers are to the lead vehicle, the harder they will brake and the less they will attempt to avoid crashing by turning the steering wheel. Because of the difficulty to implement real-world protocols, very few studies have compared real-world or on-road driving with drivers’ performances on motion based simulators. Ekanayake et al. [12] observed no differences in behavior between driving on an open road, a track or a simulator. However, the type of environment (few or many potential hazards on the road), was found to affect drivers’ speed, steering wheel angle, gas consumption and braking, with vehicles of all kinds. All these previous findings suggested that it is necessary to assess the validity of simulators based on drivers’ behavior when confronted with a hazard.

#### Physiological reactions

A dangerous situation, as a road hazard, elicits reactions of fear and stress, which, by activating the sympathetic system [13], are reflected at the physiological level by an increase in the heart rate (HR), breathing rate (BR) and sweat production rate [14,15]. Despite the difficulties of carrying out physiological measurements in real situations on open roads, some authors observed that driving in crowded downtown areas is held to be more stressful than driving on a portion of the highway during off-peak hours [16–19]. They reported a positive correlation between heart rate and EDA responses and the number of potentially hazardous situations in the driving environment and specified that overtaking and hard braking are the most stressful situations for older drivers. On the one hand, some other authors recorded similar results in driving simulators to those obtained in the real world in terms of an increase in the heart rate, Low Frequency band/High Frequency band ratio (LF/HF ratio), breathing rate and EDA in various stressful situations [20–22]. On the other hand, very few studies are available to our knowledge in which driving a real vehicle is compared with driving in a simulator, and the results obtained on these lines are rather patchy. Mueller et al. [23] recorded identical heart rates regardless of the type of vehicle (real or simulated) involved, whereas Engström, Johansson, & Östlund [24] and Johnson et al. [25] reported that an increase in heart rate and EDA occurred during stressful events only in real vehicles, but no breathing rate differences were observed. In addition, due to large differences in the risk levels induced in studies performed solely on simulators, it is not possible to reach any definite conclusions about the physiological validity of driving simulators in stressful situations [26]. All in all, the effects of stress on drivers’ physiological parameters do not seem to be influenced by the driving time, but Schneegass et al. [18] noted that the evolution of EDA in a prolonged stressful situation is only observable during the first minute, and Johnson et al. [25] detected clear-cut variations in heart rate upon observing only the first 15 seconds after the event.

#### Simulator limitations

The heterogeneity of the procedures used so far is not the only barrier to studies on the validity of simulators. On the one hand, simulator sickness is another aspect which has been widely studied in the field of virtual reality. Although this problem can be minimized by adapting factors such as the type of cabin [27] or the dynamics of the simulations [28], it is still an almost ubiquitous problem in the field of simulation, which can be increased by the occurrence of stress [29] or a strong feeling of presence [30]. The possibility of measuring this process should help to understand its influence and its interactions with all the parameters of interest, since it can influence drivers’ performances [31] and their physiological state in terms of parameters such as the heart rate [32].

On the other hand, when studying responses to danger, one has to opt between creating a surprise effect in a single initial trial [33] or recording several events and averaging the effects [34]. Drivers learn from experience, and adapt their driving behavior by acquiring new skills [2,35]. An intermediate solution may consist in triggering several unexpected hazardous events and analyzing them independently of each other.

#### Aim of the study

The aim of this study was to investigate more closely the validity of a dynamic simulator (Shepa^2^) from the human factors perspective, using various simulated situations resembling those previously tested in the framework of research and development with a view to comparing drivers’ behavior, in terms of both their physiological and declarative responses to a hazardous event when driving a real vehicle on a track versus a motion based simulator, during a constant-speed task. Based on the literature available so far, we hypothesized that (i) a strong feeling of presence would be observed under both real car and simulated driving conditions; (ii) relative or absolute validity of the motion based simulator would be observed in comparison with real car driving, in terms of the drivers’ behavioral and physiological parameters; (iii) a decrease in the strength of the driving and physiological responses would be observed between the first and second occurrence of an unexpected hazardous event.

## Methods

The study was approved by the “Comité d’hygiène, de sécurité et des conditions de travail” (Hygiene, Safety and Working Conditions Committee) from Groupe PSA and the place where the research was carried out was validated as biomedical research site n°15–225 by the “Agence Régionale de Santé - Ile de France” (regional health agency Ile de France). A written consent form was read and signed by each participants. The individual in this manuscript has given written informed consent (as outlined in PLOS consent form) to publish these case details.

### Vehicles and the environment

**Fig 1:**
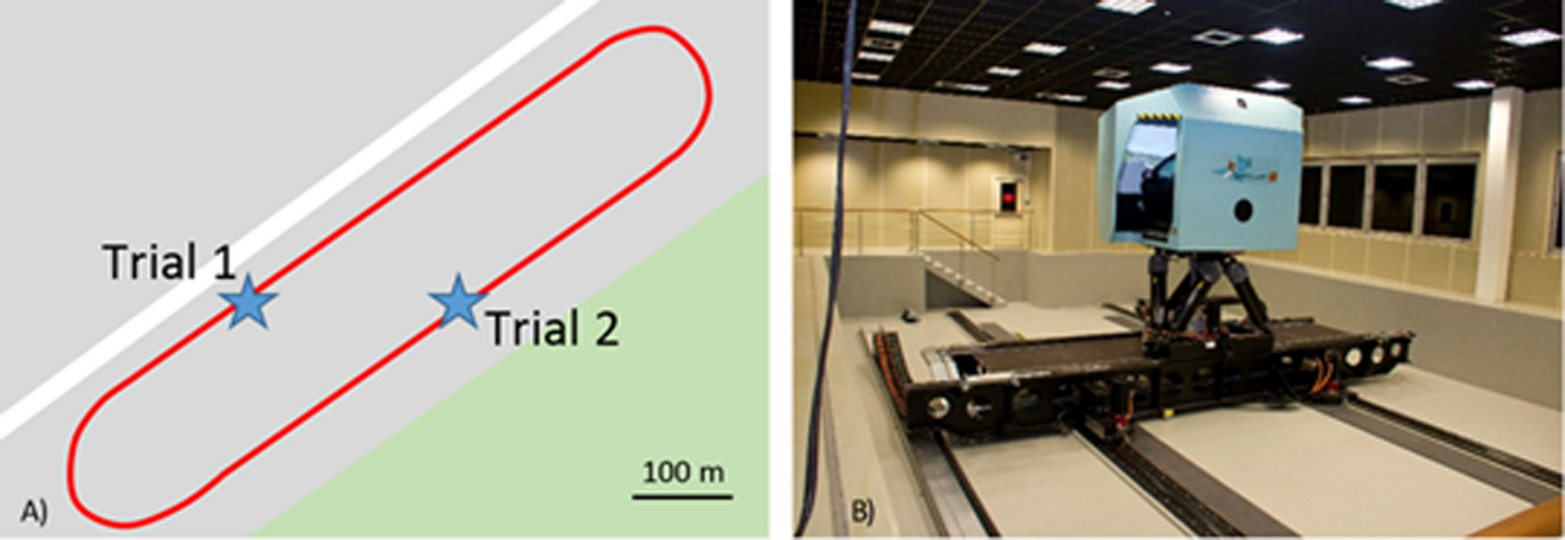
A. The Sherpa2 driving simulator. B. The map of the circuit. In red, the loop circuit used by the drivers. Blue stars give the positions of the gym balls crossing the driver’s path at kilometer 6 (trial 1) and kilometer 12 (trial 2).

The real car used in this study was a Citroën C3 with a manual gearbox. The simulated car was a dynamic model of the same car. The driving simulator was the Sherpa2 (see Fig 1A) available at the technical center of the Groupe PSA in Vélizy (France). The motion system was composed of a hexapod placed on an X-Y platform measuring 10m by 5.5m with 8 degrees of freedom. The cabin was a fully equipped half-cab Citroen C1 fitted with three screens measuring 2m x 1.2m covering a field of view of 160°, placed 2.1m from the driver’s eyes. Inside the simulation cabin, the three projection screens had a resolution of 2560x1600 each, working at a sampling frequency of 120Hz with a maximum illumination of 2500 lumens. The sounds perceived inside the vehicle were rendered using a surround system. The road was a private circuit loop approximately 1550 m in length (see the red line in Fig 1B), which was visible in the simulated environment.

### Procedure

**Fig 2:**
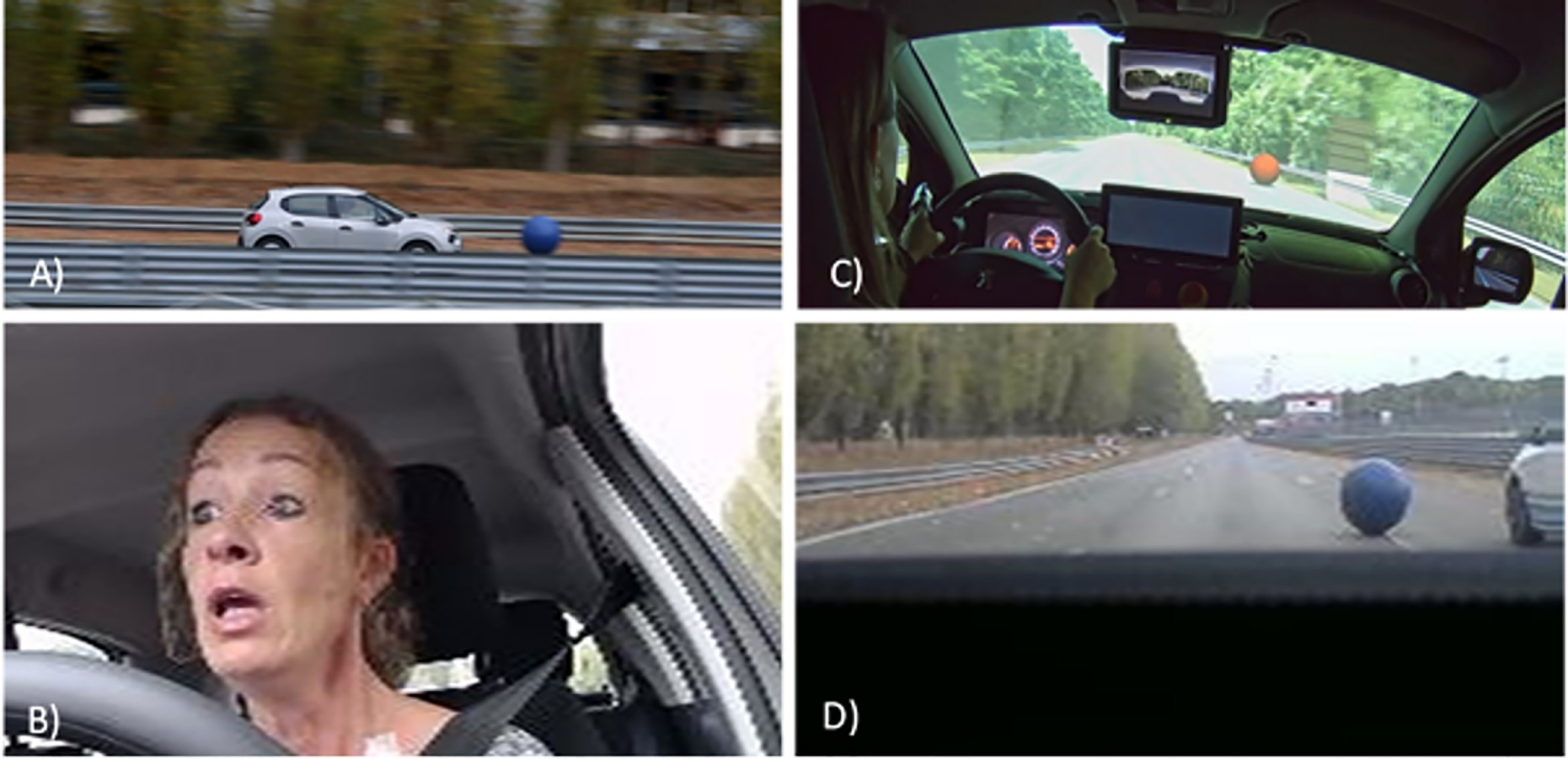
Protocol images. A) The car on the circuit with the ball crossing its path. B) The facial reaction of a driver discovering the ball crossing the road. C) A driver in the simulator with the ball beginning to cross the vehicle’s path. D) A view of the ball crossing the road as seen from inside the real car.

The experiment began by briefly describing the test (the equipment, driving conditions and questionnaires used). The drivers were equipped for undergoing electro-cardiogram (ECG), breathing and electro-dermal conductance (EDA) measurements and informed about filling in the Simulator Sickness Questionnaire (SSQ) [36]. Next, they were installed in the vehicle (a real car or a driving simulator) and asked to just sit there for 5 minutes so that baseline measurements of the physiological data could be obtained. They then had to drive 10 rounds on the 1.5-kilometer loop for about 20 minutes, keeping their speed at 55, 60, 65 or 70 kph, depending on the instructions given by a pre-recorded voice, which changed the speed limit every kilometer. At kilometer 6 and kilometer 12, the participants were keeping a speed of 60 kph when an unexpected gym ball with a diameter of 1 meter suddenly crossed the road just ahead of the vehicle (For an example of a ball crossing the road and driver’s reaction, see Fig 2). After the run, they filled in the SSQ, the National Aeronautics and Space Administration—Raw Task Load Index (NASA-RTLX) [37–39] and the Measurement Effect Conditions—Spatial Presence Questionnaire (MEC-SPQ) [38].

### Participants

One group of 28 participants (14 women: mean age = 53, SD = 7.5; 14 men: mean age = 50, SD = 7.3) and another group of 13 participants (7 women: mean age = 39, SD = 12.4; 6 men: mean age = 49, SD = 5.3) were recruited. They had all obtained their driving license more than 10 years previously (mean no. of years holding a driving license: 28 years, SD = 10.6), and they all signed an informed consent form and a safety questionnaire. They were informed that they could leave the experiment at any time. The first group participated in a real car driving test (G1R) followed by a simulated driving test (G1S) and the second group participated only in a simulated driving test (G2S).

For technical reasons, the first group drove first on the circuit and a few weeks later in the driving simulator. The sessions were presented to the participants as two independent studies to preserve the unexpected effect of the ball’s arrival. In addition, in order to make sure that the lack of counter-balancing would not bias the results, the second group, which had to drive only under simulated conditions, was used as a control group.

## Measurements and data processing

### Questionnaire data

#### Simulator sickness questionnaire

The Simulator Sickness Questionnaire (SSQ), which was filled out both before and after each driving session, contains sixteen questions about personal presence which commonly accompany simulation sickness. Here we used the Canadian-French version of the questionnaire translated by Québec University’s Cyberpsychology Laboratory in Outaouais, as suggested by Kennedy, Lane, Berbaum, & Lilienthal [36]. Each participant’s global score and the three subscale scores were calculated. The ’Nausea’ score was based on answers to questions about physiological discomfort, the ’Oculo-motor’ score was based on answers to some of the questions about oculo-motor fatigue, and the ’Discomfort’ score was based on answers to some of the questions about the participants’ spatial perception of their body.

#### NASA-RTLX

The NASA-Raw Task Load Index (NASA-RTLX) questionnaire, which was filled out after the driving session, is a six-dimensional questionnaire (mental demand, physical demand, temporal demand, performance, effort and satisfaction) designed to evaluate a posteriori the subjective workload exerted by a task. Each rating scale ranges from 0 to 20. The scores obtained on each question were kept for subsequent analysis.

#### MEC-SPQ

**Fig 3:**
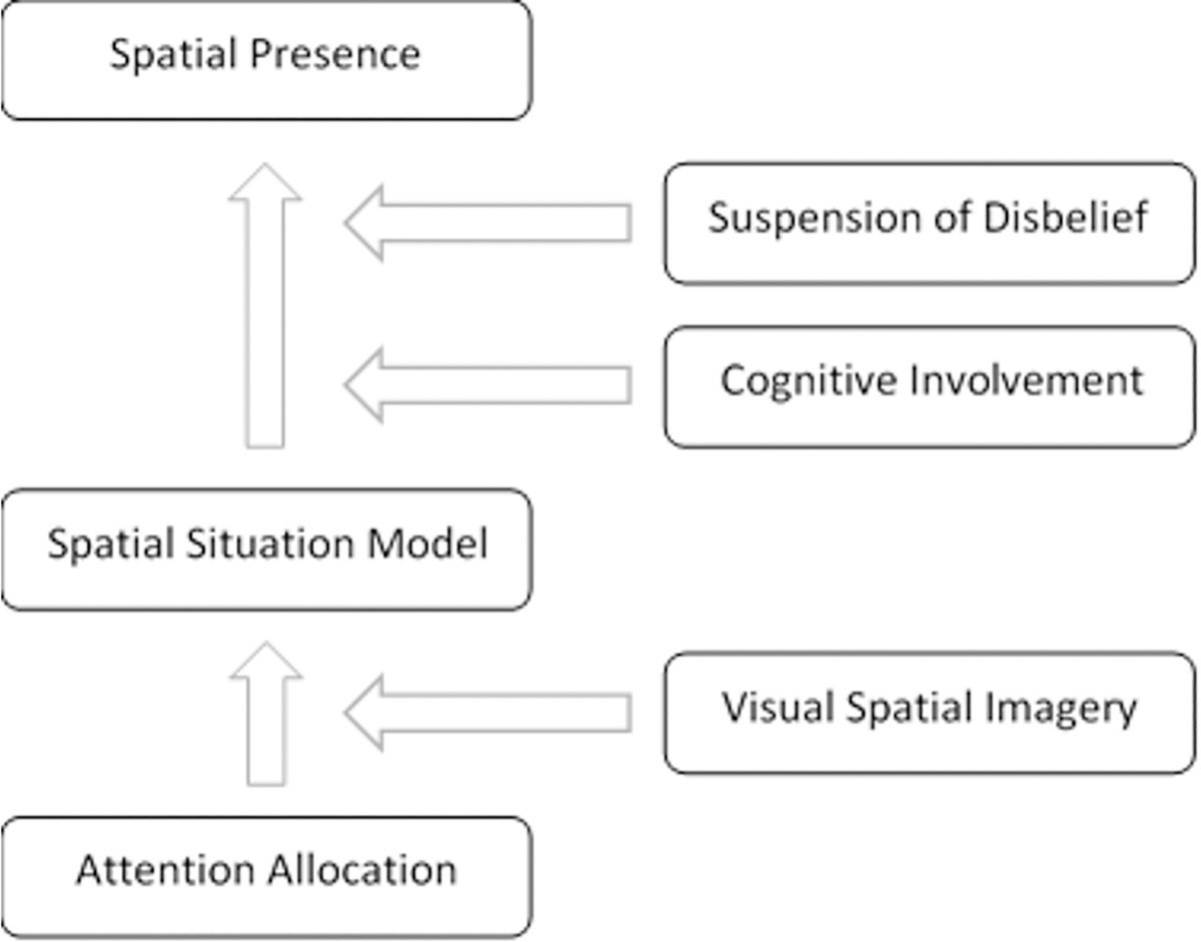
Our version of the MEC questionnaire adapted from Vorderer et al. [38].

The Measurement Effect Condition—Spatial Presence Questionnaire (MEC-SPQ) is based on the model presented by Wirth et al. [40], which was first applied by Vorderer et al. [38] (see Fig 3). For the purpose of our experiments, it was translated into French by Deniaud, Honnet, Jeanne, & Mestre [41]. One of the main advantages of this questionnaire is that it can be used in both virtual and real environments.

The version of the questionnaire used here, which comprised 6 categories (see Fig 3), reflects the two-level model for the formation of Spatial Presence [40]. Each category corresponds to 4 questions, which were presented in randomized order. The average score was calculated for each category from a 5-point Likert scale (ranging from “I don’t agree” to “I fully agree”).

The first level in the model corresponds to the “Spatial Situation model” (SSM) category. It is based on the idea that the participants build a mental spatial model of the situation with which they are faced. This category corresponds to questions such as “I had an exact idea of the spatial surroundings”. SSM depends on “attention allocation” (AA) (“I devoted my whole attention to the task”) and “visual spatial imagery” (“I can easily imagine a space without being there”).

SSM is a prerequisite for the second level, “Spatial Presence: Self location” (SPSL) (“I felt as if I was actually part of the environment”). Spatial Presence corresponds to two categories: “Suspension of Disbelief” (SoD) (“I did not pay any attention to inconsistencies in the environment”) and “Cognitive Involvement” (CI) (“the environment drove my thinking”).

### Driving data

Driving data were recorded in both vehicles (the real car and the simulator) to obtain speed, acceleration, steering wheel angle, rotation of the engine per minute (rpm) and GPS position data. The sampling frequency was set at 100 Hz. A 30-sec window, beginning 10 seconds before and ending 20 seconds after crossing the points at which the events occurred (see the blue stars in Fig 1B) was computed for each participant on the rounds in which the event occurred (with the ball), as well as on the previous rounds (no ball). The mean score and the variability of each window were calculated for each participant and each of the driving parameters.

### Physiological data

Drivers were equipped with Bionomadix devices connected to a Biopac MP160 data acquisition hardware with a 2000-Hz sampling rate. In line with classical recommendations, electrocardiogram (ECG) was recorded with three electrodes placed on the chest, skin conductance (EDA) with two electrodes placed on the left index and middle finger, and breathing parameters with a chest belt sensor. First, the data were normalized based on a 5-min pre-driving rest period and the four 30-s windows of interest were selected. Secondly, supervised artefact removal techniques were used for cleaning and processing the data. The mean heart rate [42] was obtained with band pass filtering from 0.5 to 30 Hz, order 5 calculation of the high-squared derivative using short-period smoothing (0.5 s) and long-period smoothing (2.0 s), hysteresis filtering of the difference in smoothness, a search for peaks and the median value of 3 peaks, and any increases outside the 5–95% range were removed. The mean breathing rate was calculated with band pass filtering from 0.1 to 1 Hz, an order 5 search by quantile 25 and 75% minimum and maximum levels for hysteresis filtering, and the time elapsing between two off-trough periods was calculated. Lastly, the mean phasic EDA [43] was calculated with under-sampling at 0.5 Hz, using a 32-point frequency analysis with a 95% recovery rate, and power extraction was performed from 0.05 to 0.25 Hz. The cleaned electrocardiogram signal was processed with a Fast Fourier Transformation (FFT) to separate HRV into its component Low Frequency (LF) and High Frequency (HF) rhythms. These frequency bands fall in the 0.04–0.15 Hz and 0.14–0.40 Hz range, respectively [13]. In each 30-sec window, a LF and a HF value were retrieved and the LF/HF ratio was calculated.

### Statistical analyses

Non-parametric analyses were performed on the results of the various questionnaires, since the data were discontinuous. The global SSQ score and the scores obtained on each subscale were analyzed using a Friedman ANOVA to compare groups G1R and G2S and a Wilcoxon test was also performed to further explain 2 by 2 differences. To compare group G2S with G1S and G1R, a Mann-Whitney U test was performed. As with the NASA-RTLX and the MEC-SPQ, the total score and the scores obtained on each dimension were analyzed with a Wilcoxon test to compare G1R and G2S, and Mann Whitney U-tests were used to compare G2S with G1S and G1R.

Drivers’ behavior in response to the unexpected arrival of the ball was categorized based on the criteria presented in Table 1.

**Table 1:** Classification of drivers’ behaviors in the situation with the ball crossing the road.

	Maximal absolute longitudinal deceleration	Maximal absolute lateral acceleration
No reaction	< 1 m/s^2^	< 2 m/s^2^
Braking	> 1 m/s^2^	< 2 m/s^2^
Steering	< 1 m/s^2^	> 2 m/s^2^
Braking and steering	> 1 m/s^2^	> 2 m/s^2^

To compare the results obtained by groups G1R and G1S, a repeated measures ANOVA was also performed with three intra effects: vehicle (real car *vs* simulator), trial (trial 1 *vs* trial 2) and ball (with *vs* without the ball) was performed. To obtain control data without any prior exposure to the experiment, G2S was compared with G1R and again separately with G1S by performing a repeated measures ANOVA using an inter effect: group (G1R or G1S *vs* G2S) and two intra effects: trial (trial 1 *vs* trial 2) and ball (with *vs* without the ball). In all these statistical tests, the first and second levels of interaction were calculated and a post-hoc analysis was performed using a Bonferonni test. With each of these effects, the highest levels of interaction observed are presented here in the case of significant results.

All the statistical analysis were performed using Statistica software® v.10 (Statsoft Inc. France). Data are expressed as means ± SEM.

## Results

### Questionnaires

#### NASA-RTLX (Fig 4)

The statistical analyses indicated only that G1S obtained a higher score on ’Physical demand’ than G2S (≈ +3.0 points, *p*<0.01). No other significant differences were observed in any of the global NASA-RTLX scores or subscale scores between G1R, G1S and G2S.

**Fig 4:**
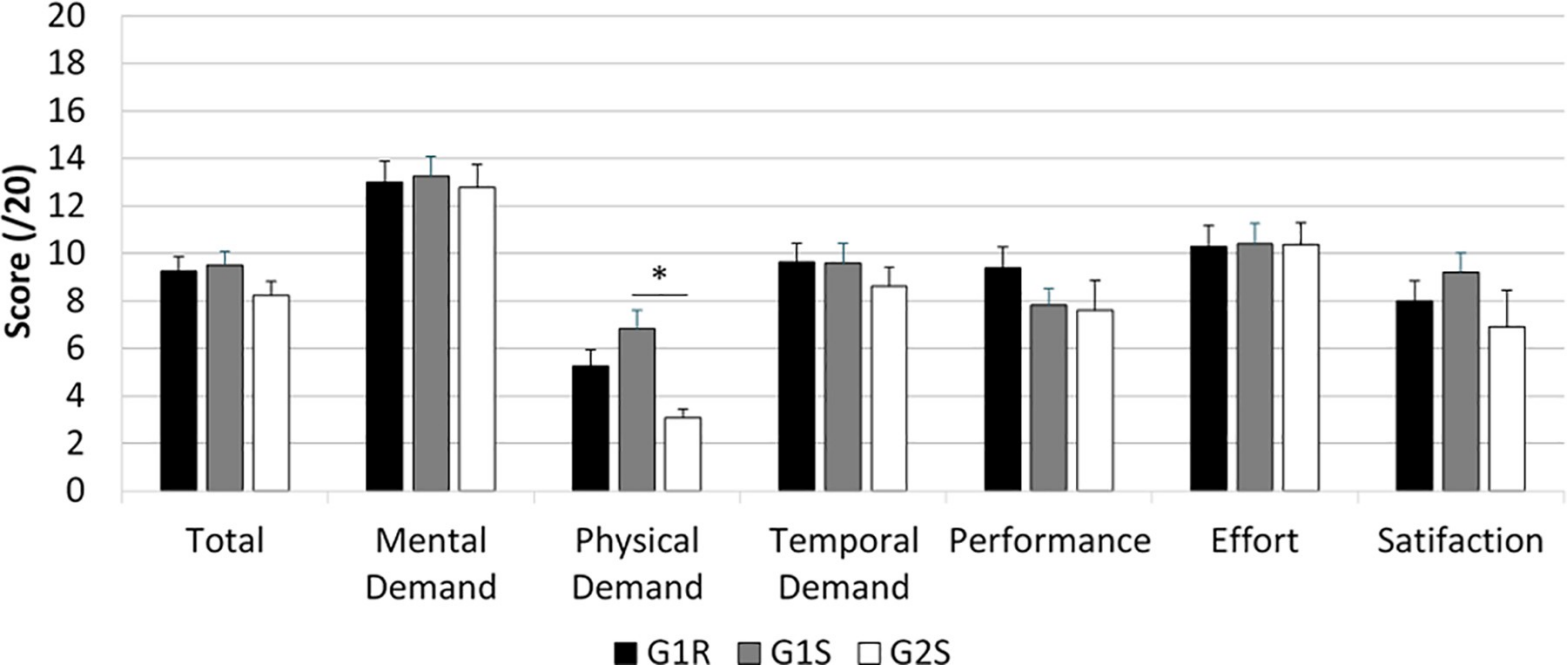
The NASA-RTLX scores.

#### Simulator sickness questionnaire (Fig 5)

As regards the evolution of the simulator sickness symptoms, the statistical analysis indicated that the G1R score on ’Nausea’ was higher after than before the driving session (≈ x2, *p*<0.05). G1S gave a higher ’Global’ score (≈ x3, *p*<0.001) after than before the driving session, which was also the case with the various subscales [’Nausea’ (≈ x3, *p*<0.01), ’Oculo-motor’ (≈ x3, *p*<0.001), and ’Disorientation’ (≈ x3, *p*<0.01)]. G2S gave a higher ’Global’ score (≈ x2, *p*<0.05) after than before the driving session, which was also the case with ’Nausea’ (≈ x3, *p*<0.05).

**Fig 5:**
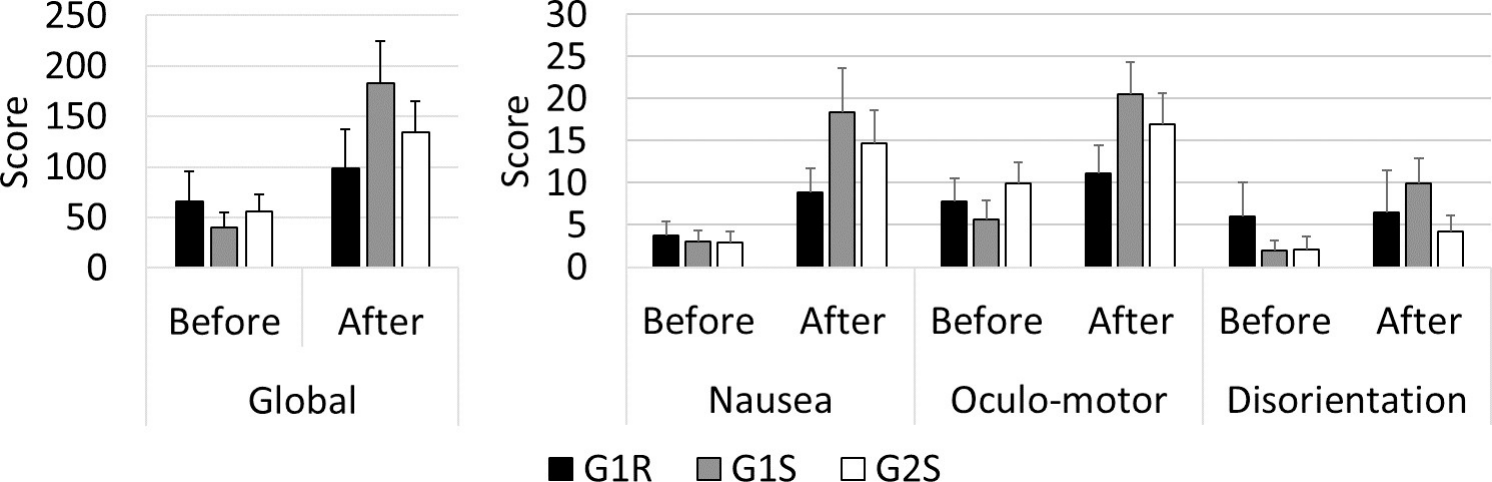
The SSQ scores. On the left, the global score; on the right, the details of the subscale scores.

Comparisons between the various groups indicated that after the driving session, G1S gave higher ’Global’ (≈ x2, *p*<0.05), ’Nausea’ (≈ x2, *p*<0.05), and ’Oculo-motor’ scores (≈ x2, *p*<0.05) than G1R. No significant differences emerged from the G1S versus G2S comparisons or from the G1R versus G2S comparisons.

During the driving session, group G1R showed an increase in nausea; G2S gave higher global and nausea scores; and G1S gave a higher global score and higher SSQ subscale scores.

#### MEC-SPQ (Fig 6)

The statistical analyses indicated that G1R obtained significantly lower scores than G1S on the ’Spatial Situation model’ (SSM) (≈ -0.5 point, *p*<0.01), ’Suspension of Disbelief’ (SoD) (≈ -0.5 point, *p*<0.05) and ’Spatial Presence: Self Location’ (SPSL) (≈ -0.5 point, *p*<0.05).

**Fig 6:**
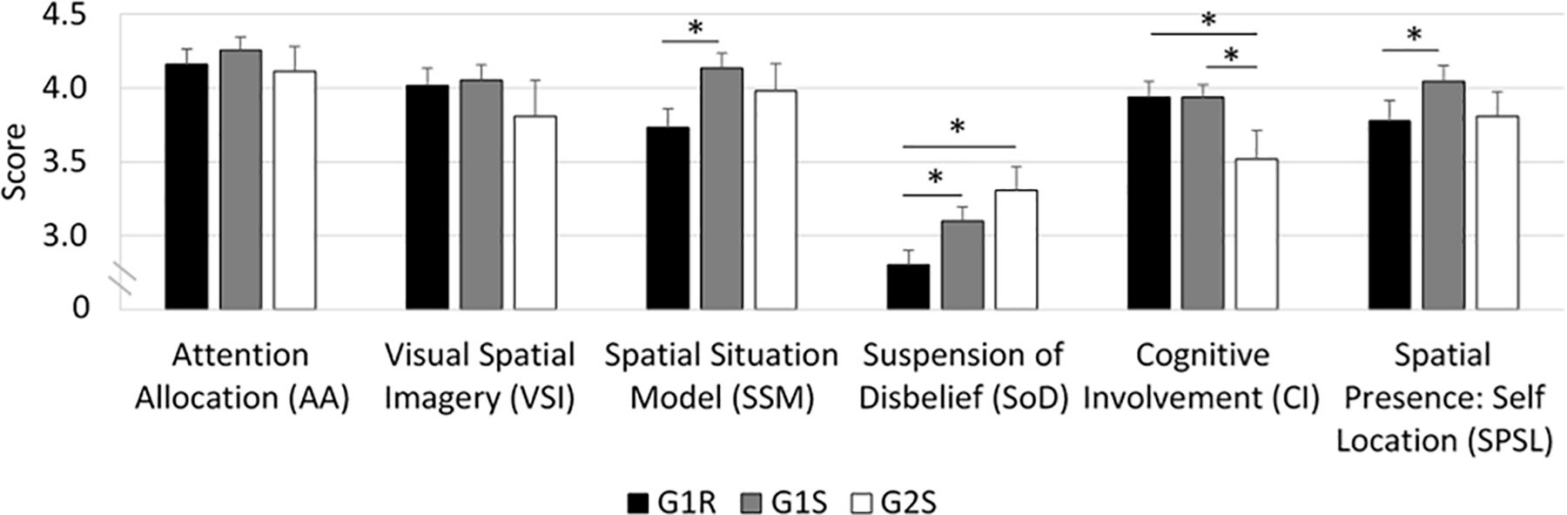
MEC-SPQ scores obtained by each group.

The comparisons between G1R and G2S indicated that G1R obtained lower scores than G2S on ’Suspension of Disbelief’ (SoD) (≈ -1 point, *p*<0.05) and ’Cognitive Involvement’ (CI) (≈ -0.5 point, *p*<0.05).

The comparisons between G1S and G2S indicated that G1S obtained significantly higher scores than G2S on ’Cognitive Involvement’ (CI) (≈ +0.5 point, *p*<0.05).

To summarize the results obtained on the “Presence” questionnaire, G1R obtained lower SSM, SoD and SPSL scores than G1S; G1S obtained a lower SoD score than G2S; and G1R and G1S obtained higher CI scores than G2S.

### Driving data

#### Driving reactions in response to the ball (Fig 7)

When faced with the ball crossing the road, drivers produced different responses which we placed in four categories: ’no reaction’, ’braking’, ’steering’ and ’braking and steering’ (see Table 1). This classification helped to understand more clearly the driving results presented below by providing an overview of drivers’ reactions in situations involving unexpected obstacles. The main finding which emerged here was that only trial 2 performed by group G2S yielded a different pattern of reaction from the other trials, including a high rate of braking behavior (76%) and no steering responses, whereas other types of response, except for the absence of reaction, accounted for between 10% and 50%. An evolution was also observed in the case of G1R, where fewer steering actions and more ’brake and steer’ responses were observed in trial 2 than in trial 1.

**Fig 7:**
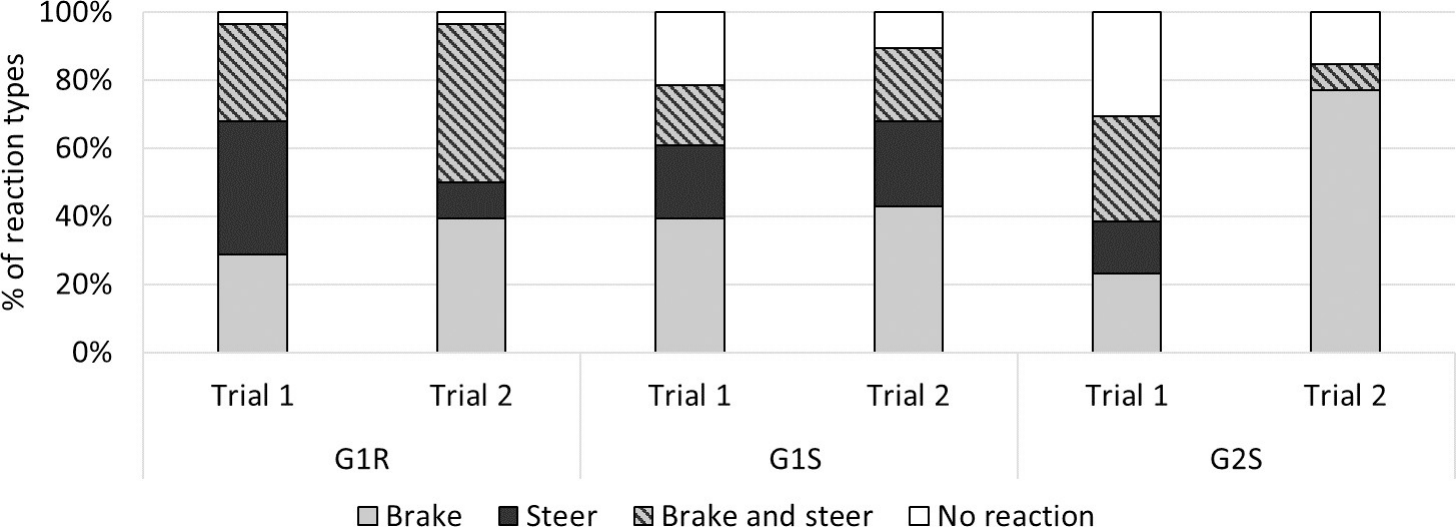
Driving behavior observed in response to the ball crossing the road in front of the vehicle.

#### Speed (Fig 8)

Comparisons between groups G1R and G1S showed that there was a main effect of ’ball’ (*F*_(27,1)_ = 12.78, *p*<0.01). The Bonferonni post-hoc analysis showed that drivers had a lower mean speed in the situation with the ball than without the ball (≈ -4 kph, p<0.01). In addition, a significant interaction was found to occur between ’vehicle’ and ’trial’ (*F*_(27,1)_ = 4.96, *p*<0.05). The post-hoc analysis indicated that G1R had a lower mean speed than G1S in trial 2 (≈ -4 kph, *p*<0.001).

**Fig 8:**
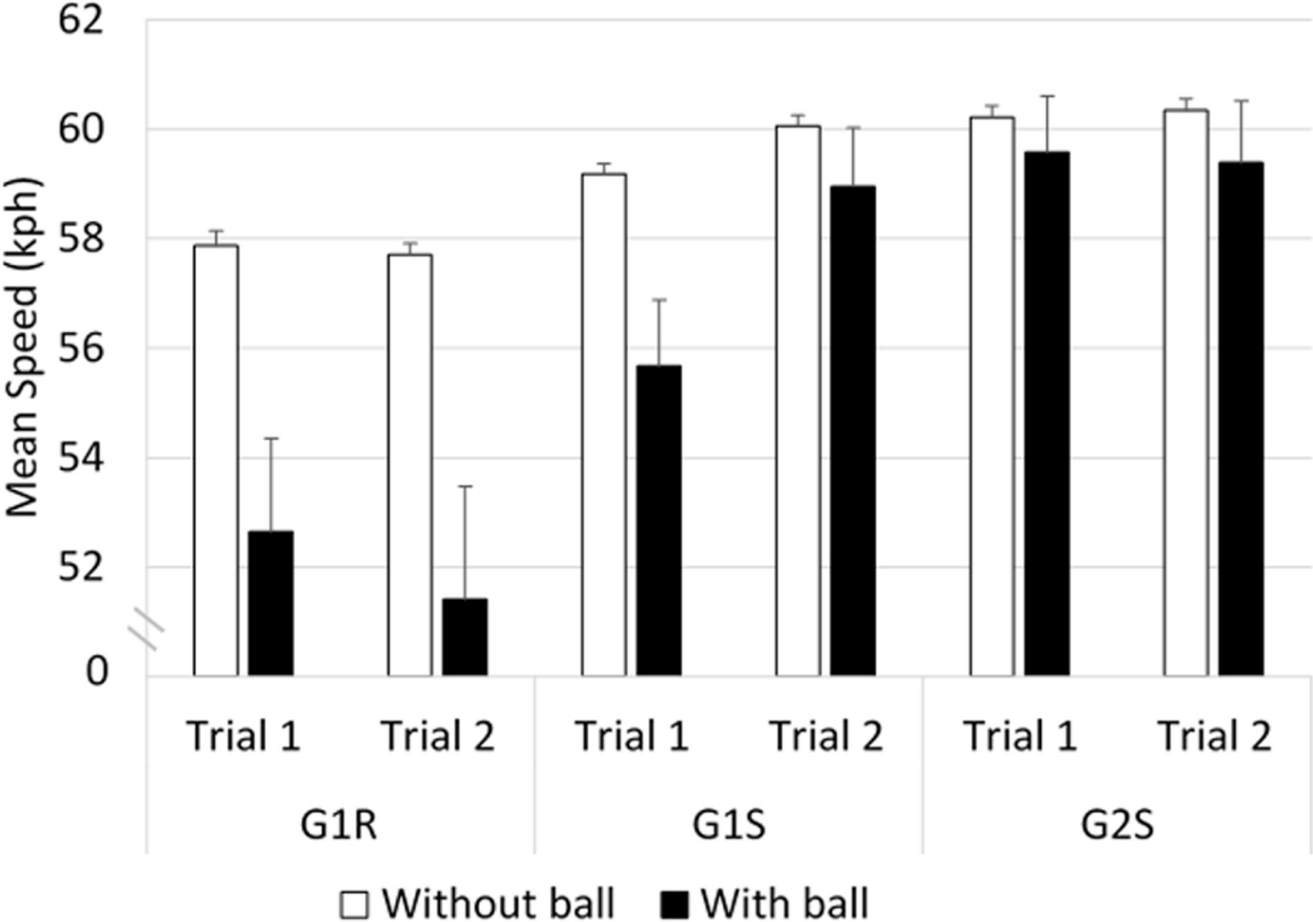
Mean speed displayed on the speedometer.

Comparisons between groups G1R and G2S showed the existence of a significant interaction between ’ball’ and ’group’ (*F*_(39,1)_ = 4.17, *p*<0.01). The post-hoc analysis showed that in the case of G1R, the mean speed was greater in the situation without the ball than with the ball (≈ +6 kph, *p*<0.001). In the situation with the ball, G1R drove more slowly than G2S (≈ -7 kph, *p*<0.001).

No significant differences were observed between G1S and G2S in terms of the speed.

To sum up the speed behavior, groups G1R and G1S drove more slowly in the situation with the ball than without the ball; and in the situation with the ball, G1R drove more slowly than G2S.

#### Deceleration (Fig 9)

As regards the mean deceleration, comparisons between G1R and G1S showed the occurrence of a significant interaction between ’trial’ and ’ball’ (*F*_(27,1)_ = 8.30, *p*<0.01). The Bonferonni post-hoc test showed that in trial 1, the drivers decelerated more strongly with than without the ball (≈ +0.3 m.s^-2^, *p*<0.001), and likewise in trial 2 (≈ +0.5 m.s^-2^, *p*<0.001) with the ball, that they performed less deceleration in trial 1 than in trial 2 (≈ -0.2 m.s^-2^, *p*<0.01).

**Fig 9:**
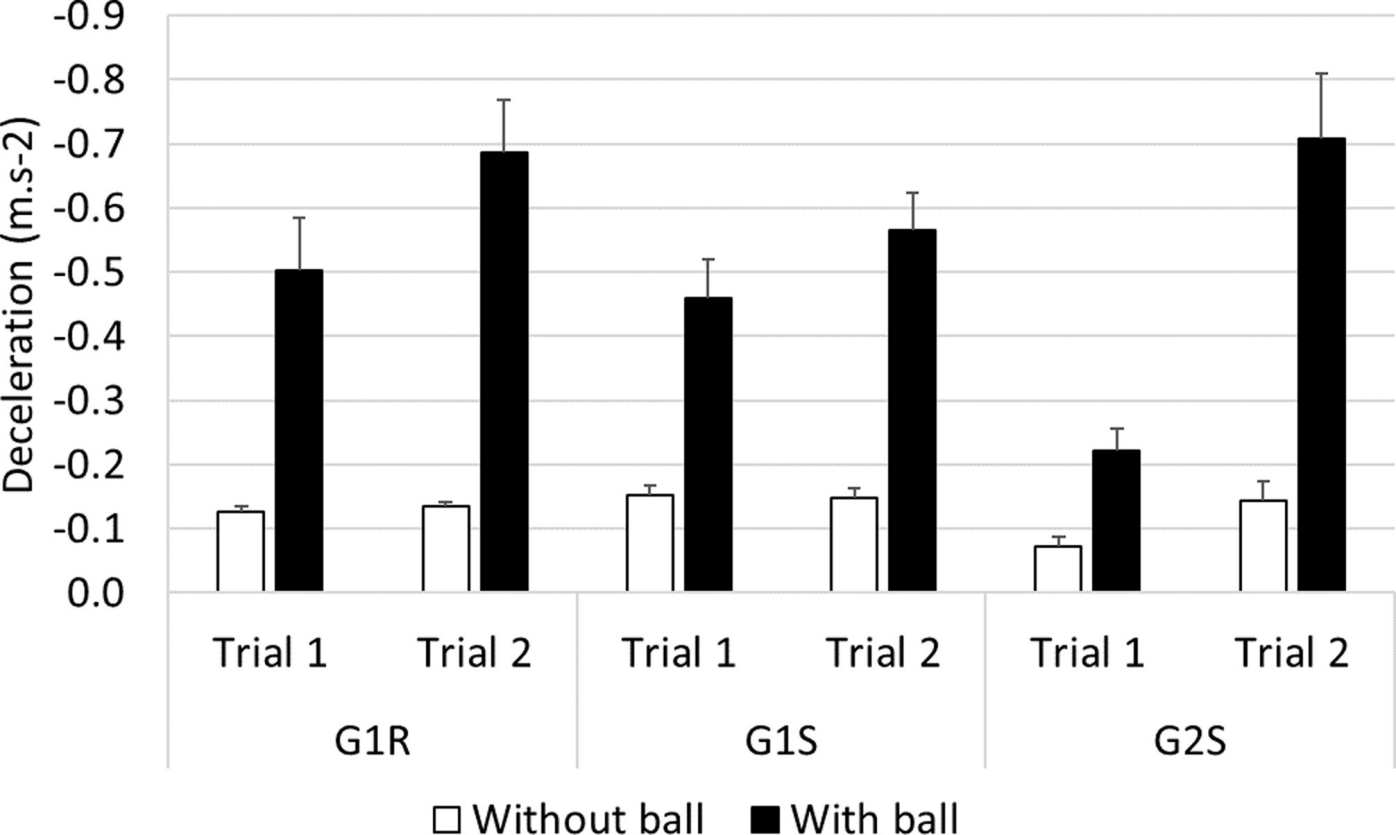
Deceleration of vehicles, based on the mean speed variability.

Based on the comparisons between G1R and G2S, a significant interaction can be said to have occurred between ’group’ and ’trial’ (*F*_(39,1)_ = 6.66, *p*<0.05). The Bonferonni test indicated that in the case of G2S, less deceleration occurred in trial 1 than in trial 2 (≈ -0.25 m.s^-2^, *p*<0.01). In addition, an interaction was observed between ’trial’ and ’ball’ (*F*_(39,1)_ = 16.15, *p*<0.001). The post-hoc analysis showed that in the situation with the ball, less deceleration occurred in trial 1 than in trial 2 (≈ -0.35 m.s^-2^, *p*<0.001) and lastly, that greater acceleration occurred in the situation with the ball than without the ball in both trial 1 (≈ +0.25 m.s^-2^, *p*<0.001) and trial 2 (≈ +0.55 m.s^-2^, *p*<0.001).

In the comparisons between groups G1S and G2S, the ANOVA showed the occurrence of a significant interaction between ’group’ and ’trial’ (*F*_(27,1)_ = 15.08, *p*<0.001). The main results obtained in the post-hoc analysis showed that in the case of G2S, less deceleration occurred in trial 1 than in trial 2 (≈ -0.25 m.s^-2^, *p*<0.01). In addition, an interaction was observed between ’trial’ and ’ball’ (*F*_(39,1)_ = 23.69, *p*<0.001). The post-hoc analysis indicated that in the situation with the ball, less deceleration occurred in trial 1 than in trial 2 (≈ -0.3 m.s^-2^, *p*<0.001). Lastly, greater deceleration occurred in the situation with the ball than without the ball in both trial 1 (≈ +0.2 m.s^-2^, *p*<0.001) and trial 2 (≈ +0.5 m.s^-2^, *p*<0.001).

The main results obtained on the participants’ braking behavior indicated that the deceleration was greater in the condition with the ball than without the ball in all the groups; the drivers in all the groups decelerated more strongly at their second exposure to the ball crossing the road.

#### Steering wheel angle (Fig 10)

Comparisons between groups G1R and G1S showed that the absolute steering wheel angle was influenced by an interaction between ’vehicle’ and ’trial’ (*F*_(27,1)_ = 9.31, *p*<0.01). The main results of the post-hoc analysis indicated that in the case of G1R, the values recorded were lower in trial 1 than in trial 2 (≈ -0.9°, *p*<0.05) and that G1R obtained a lower score in this respect than G1S in trial 1 (≈ -1.3°, *p*<0.001). In addition, an interaction was observed between ’vehicle’ and ’ball’ (*F*_(27,1)_ = 12.92, *p*<0.01). The Bonferroni post-hoc test showed that in G1R, the situation with the ball triggered larger steering wheel angles (avoidance maneuvers) than the situation without the ball (≈ +2.4°, *p*<0.001. In the situation without the ball, the steering wheel angles were smaller in G1R than in G1S (≈ -1.4°, *p*<0.001).

**Fig 10:**
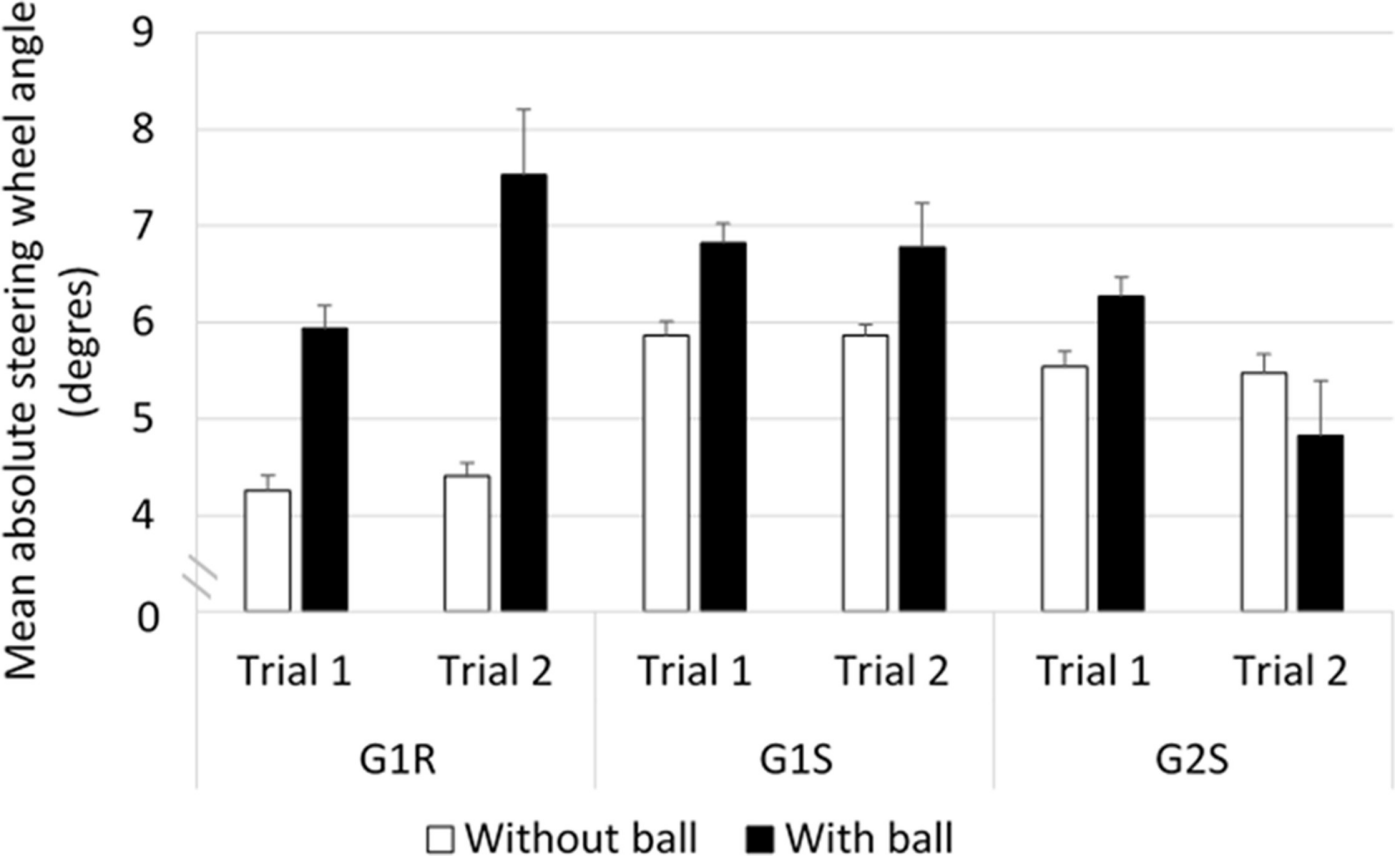
Mean absolute steering wheel angle.

In the comparisons between G1R and G2S, an interaction was observed between ’group’ and ’trial’ (*F*_(39,1)_ = 7.40, *p*<0.01). The Bonferonni post-hoc test showed the existence of no significant differences in this respect. In addition, an interaction was observed between ’group’ and ’ball’ (*F*_(39,1)_ = 18.61, *p*<0.001). The post-hoc test showed that in the case of G1R, the situation with the ball triggered larger steering wheel angles than the situation without the ball (≈ +2.4°, *p*<0.001).

In the comparisons between G1S and G2S, the statistical analysis showed that there was a main effect of ’group’ (*F*_(39,1)_ = 4.66, *p*<0.05). The post-hoc analysis showed that the absolute steering wheel angle was larger in the case of G1S than in that of G2S (≈ +0.7°, *p*<0.05). In addition, a main effect of ’trial’ was observed (*F*_(39,1)_ = 5.39, *p*<0.05). The Bonferroni post-hoc analysis showed that the absolute steering wheel angle recorded was larger during trial 1 than trial 2 (≈ +0.5°, *p*<0.001).

Based on the absolute steering wheel angle, the lateral trajectory of G1R was therefore more pronounced in the situation with the ball than without the ball crossing the road; G1S produced more lateral variations than G2S in both conditions than G1R in the condition without the ball; in the real car (G1R), drivers showed greater lateral deviations in trial 2 than in trial 1, while the opposite result was obtained in the driving simulator (in G1S and G2S).

### Physiological data

#### Heart rate (Fig 11)

In the comparisons on the mean heart rate comparisons between G1R and G1S, an interaction was observed between ’vehicle’ and ’ball’ (*F*_(27,1)_ = 51.23, *p*<0.001). The post-hoc analysis indicated that G1R showed a higher mean heart rate in the situation with the ball than without the ball (≈ +5 beats per minute, *p*<0.001). In addition, in the situation with the ball, the mean heart rate was higher in G1R than in G1S (≈ +5 bpm, *p*<0.001).

**Fig 11:**
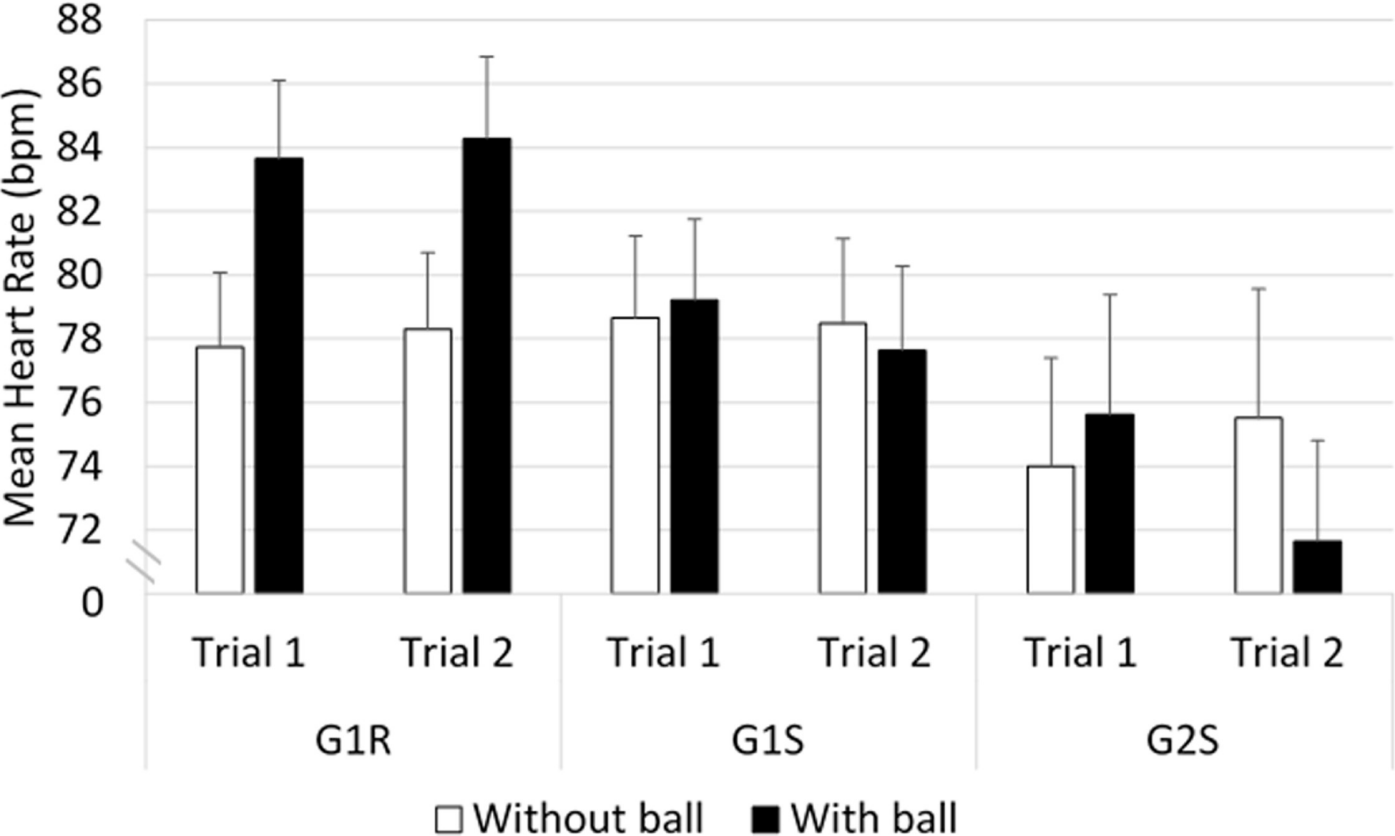
Mean heart rate.

The ANOVA conducted on the mean heart rate in G1R versus G2S indicated that an interaction occurred between ’group’ and ’ball’ (*F*_(39,1)_ = 32.32, *p*<0.001). The post-hoc analysis showed that the heart rate values were higher in G1R in the situation with the ball than without the ball (≈ +5 bpm, *p*<0.001). In addition, an interaction was observed between ’trial’ and ’ball’ (*F*_(39,1)_ = 5.50, *p*<0.05). The Bonferonni post-hoc analysis showed that the mean heart rate values were higher with the ball than without the ball in both trial 1 (≈ +4 bpm, *p*<0.001) and trial 2 (≈ +1 bpm, *p*<0.01).

The comparisons between G1S and G2S indicated that an interaction occurred between ’trial’ and ’ball’ (*F*_(39,1)_ = 11.87, *p*<0.01). The post-hoc analysis showed that in the situation with the ball, trial 1 induced higher heart rate values than trial 2 (≈ +4 bpm, *p*<0.001).

To sum up the drivers’ heart rate results, G1R showed higher mean heart rate values when the ball appeared than when there was no ball, contrary to what occurred with the simulator drivers; and both G1S and G2S showed higher mean heart rate values at the first exposure to the ball than to the second one.

#### LF/HF ratio (Fig 12)

The comparisons on the ratio between the mean low and high ECG frequencies in groups G1R and G1S showed that an interaction occurred between ’trial’ and ’ball’ (*F*_(39,1)_ = 33.06, *p*<0.001). The Bonferonni post-hoc analysis indicated that the situation with the ball induced higher ratios than without the ball in trial 1 (≈ +0.8, *p*<0.001). In the situation without the ball, lower ratios were recorded in trial 1 than in trial 2 (≈ -0.7, *p*<0.001).

**Fig 12:**
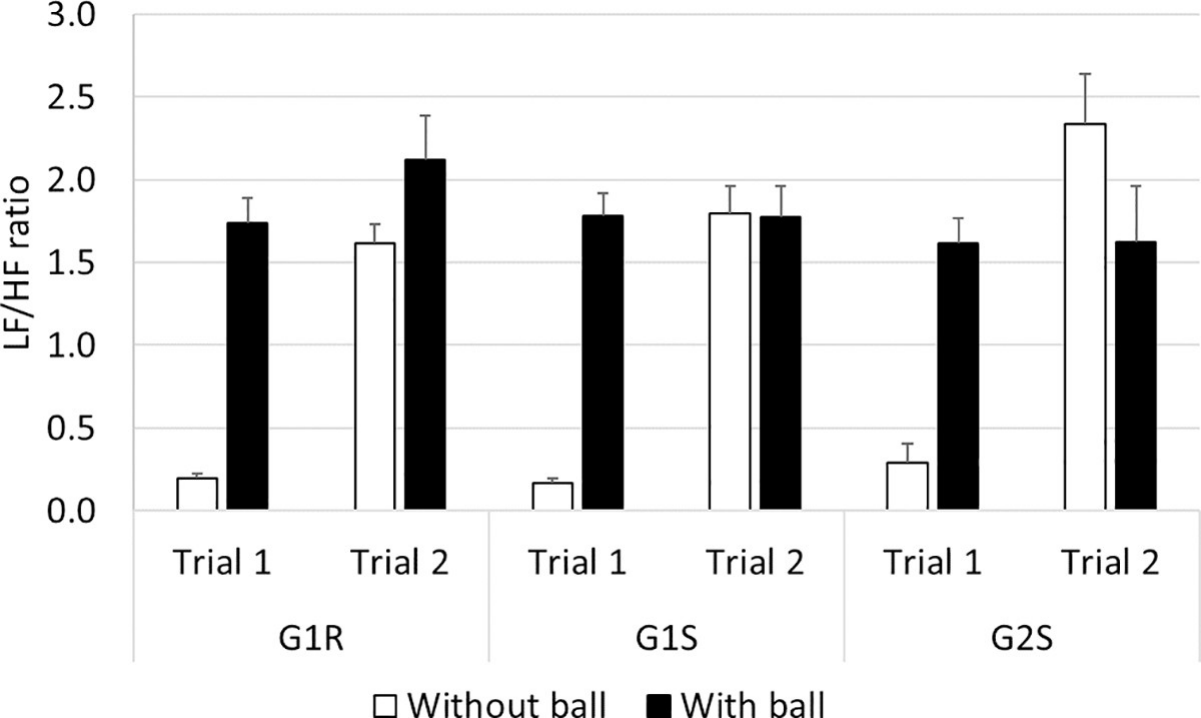
LF/HF ratio.

The comparisons between G1R and G2S showed the occurrence of an interaction between ’trial’ and ’ball’ (*F*_(39,1)_ = 28.97, *p*<0.001). The Bonferonni post-hoc analysis showed that in trial 1, higher ratios were recorded in the situation with the ball than without the ball (≈ +1.4, *p*<0.001); whereas lower ratios were recorded in trial 1 than in trial 2 (≈ -1.7, *p*<0.001) in the situation without the ball. In addition, an interaction was found to occur between ’group’ and ’ball’ (*F*_(39,1)_ = 8.37, *p*<0.001), indicating that in the case of G1R, the situation with the ball induced significantly higher LF/HF ratios than the situation without the ball (≈ +1.0, *p*<0.001).

The comparisons between G1S and G2S showed the occurrence of an interaction between ’trial’ and ’ball’ (*F*_(39,1)_ = 54.37, *p*<0.001). The Bonferonni post-hoc analysis showed that the situation with the ball induced higher values than without the ball in trial 1 (≈ +1.5, *p*<0.001), and that lower values were recorded in trial 1 than trial 2 in the situation without the ball (≈ -1.8, *p*<0.001). In addition, an interaction was found to occur between ’group’ and ’ball’ (*F*_(39,1)_ = 6.19, *p*<0.001), indicating that the situation with the ball was perceived in G1S as being more risky than the situation without the ball (≈ +0.8, *p*<0.001).

To sum up the results presented in this section, the first passage without a ball was associated with lower LF/HF ratios than any of the other passages in all the groups.

#### Breathing rate (Fig 13)

The comparisons between G1R and G1S indicated that the mean breathing rate was influenced by an interaction between ’vehicle’ and ’ball’ (*F*_(27,1)_ = 6.03, *p*<0.05). In the condition with the ball, a higher mean breathing rate was recorded in G1R than in G1S (≈ +2 breaths/min, *p*<0.01).

**Fig 13:**
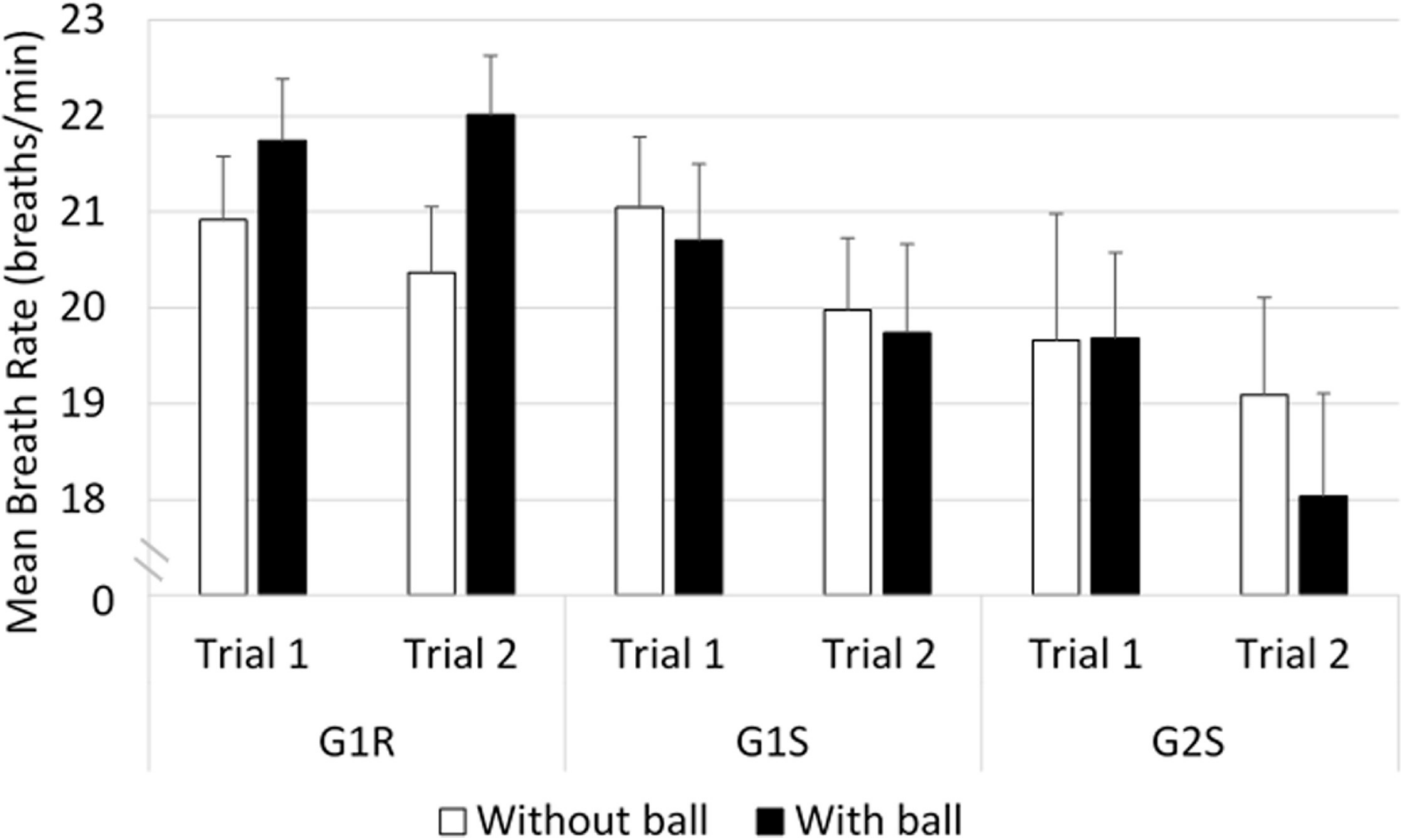
Mean breathing rate.

In the comparisons between G1R and G2S, the ANOVA indicated that there was a significant main effect of ’group’ (*F*_(39,1)_ = 4.63, *p*<0.05). A higher mean breathing rate was recorded in G1R than in G2S (≈ +2 breaths/min, *p*<0.05).

Comparisons between G1S and G2S showed the presence of a main effect of ’trial’ (*F*_(39,1)_ = 5.50, *p*<0.05). The post-hoc analysis showed that the mean breathing rate was significantly higher in trial 1 than in trial 2 (≈ +1 breaths/min, *p*<0.05).

All in all, the mean breathing rate of drivers in the real car increased in the situation with the ball; and in the simulated driving condition, the mean breathing rate was lower in trial 2.

#### Phasic EDA (Fig 14)

Comparisons on the phasic component of the EDA measurements between groups G1R and G1S indicated the presence of a significant effect of ’trial’ (*F*_(27,1)_ = 6.21, *p*<0.05). The post-hoc analysis showed that a lower phasic response was induced in trial 1 than in trial 2 (≈ -0.1, *p*<0.01) in both groups. In addition, an interaction was observed between ’vehicle’ and ’ball’ (*F*_(27,1)_ = 8.85, *p*<0.01). The post-hoc analysis showed that a higher phasic EDA response occurred in the situation with the ball than without the ball in the case of G1R (≈ +0.3, *p*<0.01). In addition, a higher phasic response was recorded in G1R than in G1S in the situation with the ball (≈ +0.3, *p*<0.001).

**Fig 14:**
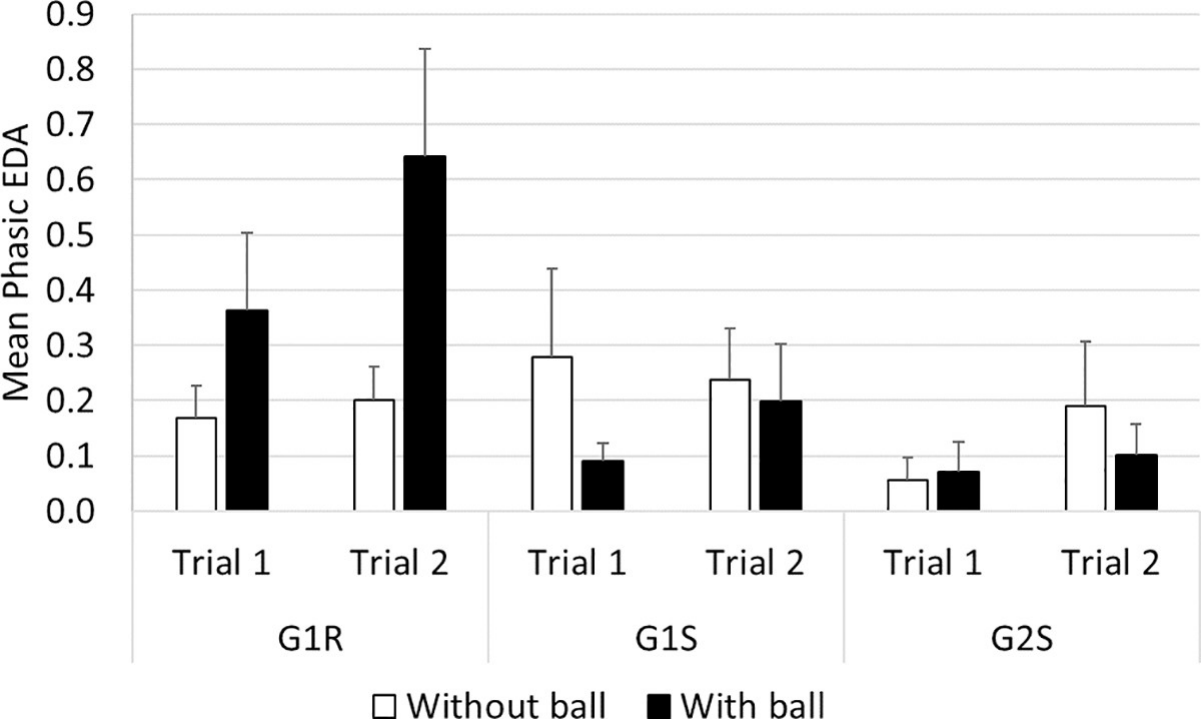
Mean phasic EDA.

No significant differences between G1R and G2S or between G1S and G2S were observed in this respect.

To resume the results obtained on the drivers’ phasic EDA responses, higher values were recorded in trial 2 than in trial 1 in both G1R and G1S; G1R showed higher values than G1S in the situation with the ball; and G1R showed higher phasic EDA responses with the ball crossing the road than without the ball.

## Discussion

In this study, the validity of drivers’ behavior in driving simulators as compared with real driving was analyzed using different indicators, including not only their driving performances, but also their declarative feeling of presence and their physiological reactions. These three aspects have been rarely studied together and even more rarely compared between a real vehicle (in this case, on a circuit) and a motion based simulator. In order investigate the use of simulators more closely, it seemed relevant to introduce a speed maintenance constraint as well as the sudden occurrence of an unexpected hazard, namely the crossing of a gym ball at a pedestrian’s fast walking speed. In order to check the reproducibility of the data obtained, each situation was tested twice in order to determine how the drivers’ behavior evolved with time. All in all, the results obtained here indicate that the workload was equivalent in all the groups tested. In the simulator, motion sickness was more severe and several sub-scales on the sense of presence were lower than in real car driving. As far as speed maintenance was concerned, the simulator showed either absolute or relative validity, depending on the driving and physiological parameters involved. An increase in the level of arousal was observed during the first encounter with the ball, which lasted until the end of the driving session under both real and simulated conditions. As regards the drivers’ reactions to the hazardous event, stronger braking occurred in the simulator than in the real vehicle, and the drivers showed stress reactions only in the real vehicle.

### Self-reported indexes to simulator validity

First we will look at the validity of the simulator via the differences and similarities observed between the drivers’ declarations while driving the real car on the track and the simulator. As previously mentioned in the SSQ, the problem of simulator sickness still remains to be solved, although none of the participants had to interrupt their tests for this reason. Although previous authors have reported that simulator sickness has deleterious effects on drivers’ performances [44], this was not the case in the present study. This may have been due to the presence of factors minimizing simulator sickness, such as a full car cabin and the motion of the car [27,28].

No differences in the reported workload were observed between driving conditions, which enhances the validity of the simulator. These results are consistent with the scores obtained on the MEC-SPQ Attention Allocation and Visuo-Spatial Imagery subscales. The Attention Allocation subscale scores did not differ significantly between groups, which suggests that the attention paid by drivers to the environment did not depend on the type of vehicle (real or simulated) involved, contrary to previous findings by Vorderer et al. [38]. As some authors have pointed out, attention allocation is a major determinant of presence [45,46]. Likewise, since participants all had similar levels of Visuo-Spatial Imagery regardless of the vehicle involved, the differences observed in the following subscales were not due to inter-individual differences in spatial projection or attention paid to the environment. In terms of Higher Cognitive Involvement, drivers in the second group obtained lower scores (-0.5 point) than those in the first group, regardless of the type of vehicle. This item may therefore be more sensitive to individuals who participated in both conditions of the study being more deeply involved than those exposed to the simulated environment alone. However, the drivers in the first group declared that they felt more clearly located in space in the virtual environment than on the circuit. A possible explanation for the latter statement may be that the drivers of the real car attempted to picture themselves in real space in a larger reference frame than in the virtual environment [47]. The circuit was certainly anchored in the real world, where the participants were able to move and locate themselves prior to the study without being familiar with this area, whereas the virtual environment is a place apart, with no access roads leading to it and no name on a map or in a GPS. The statement "I was aware of my position in the environment" can therefore be interpreted on a larger or smaller scale, depending on the context. As assumed in the Spatial Situation Model, the participants projected themselves slightly more into the virtual environment than into the real environment (+0.5 point). The fact that greater feeling of presence were elicited in the simulated environment than in the real world may seem rather surprising. But before going any further, it is worth mentioning that the circuit itself can be said to be a real but not very usual environment. There exists in fact a gap between the roads on which users drive every day and the circuit used in this experimental context. This may link up with the fact that the Suspension of Disbelief scores were lower (-0.5 point) in the real context than in the simulator, since the participants said they were less attentive to the inconsistencies of the environment in the simulator than in real life. This may be due to a tendency towards leniency when dealing with technology. The more realistic the simulated environment presented is, the fussier the users and the harder their judgements may be. Drivers may be more critical of a "semi-real" environment than of a completely simulated environment which requires either basing real vs virtual comparisons on non-declarative data or compensating for these discrepancies with more specific or complementary questions about the expected requirements of the virtual environment. This gap between reality and ’close to reality’ may link up with the ’uncanny valley’ concept, according to which the closer we get to reality, the more we see the defects. In addition, Brenton, Gillies, Ballin, & Chatting [48] have drawn a parallel between the impact of mismatching cues inducing the uncanny effect and the Break in Presence (BIP). A BIP occurs when the participant stops responding to the virtual stream and responds instead to the real sensory stream. Based on the premise that simulator sickness can be experienced as a simulation error, sick individuals tend to lose their credibility in comparison with healthy individuals [49]. Although these concepts relate to different fields, presence has to do with location and uncanny valley, with the nature of an object. This BIP creates the superimposition of two impressions in the mind that the environment is both real and not real at the same time [48]. Conversely, a simulated environment is offset enough to create a switch between feeling “I’m in a real environment” or “all of this is a simulation, I’m aware of it”, which is consistent with the Higher Cognitive Involvement answers obtained in this study.

### Validity of the simulator while simply maintaining speed or coping with road hazards

Behavioral and physiological reactions are usually triggered when drivers are confronted with a dangerous situation, consisting here of a gym ball crossing the road in front of the vehicle. It is therefore necessary to observe these responses in these specific situations in order to increase our knowledge about simulators and their reliability. In the present speed maintenance situation, the longitudinal driving parameters were identical under both real and simulated driving conditions, as observed in several previous studies at similar average speeds [5,50,51] and deceleration conditions [52]. It can therefore be concluded that in a simple driving task, the motion-based simulator showed absolute validity in terms of the longitudinal control of the vehicle. As regards the lateral control of the vehicle without any obstacles, the drivers produced a larger average absolute steering angle in the simulator (+1.5°). This finding is consistent with previous studies in which greater variability in the simulated steering angle was observed than under real-life driving conditions [53,54]. The representativeness of the steering wheel could therefore still be improved. Drivers’ difficulty in maintaining a stable trajectory may also be due, however, to the physical and oculo-motor discomfort caused by simulator sickness. The lateral management of the simulated vehicle can therefore be said to show relative validity, whereas the physiological parameters measured in the simulator under speed maintenance conditions showed absolute validity in comparison with real car driving: no differences were observed between the two types of vehicle in the heart rate, LF/HF ratio, breathing rate or phasic EDA. These results are consistent with those obtained by Li et al. [55], who reported that on a straight, wide lane road, identical heart rates were recorded in participants driving a real car versus a simulator. Johnson et al. [25] also reported that no differences in drivers’ respiratory parameters were observed between a real car and a simulator in a simple driving task. This finding is not in line with previous studies in which a higher heart rate was found to occur under real driving conditions than in a simulator [25,56]. Milleville-Pennel & Charron [29] have suggested that the presence of an observer in the car, which was also the case in the present study, cannot be an explanatory factor. The validity of this point needs to be thoroughly re-examined in the case where drivers are endangered, as we will now see.

First, the possible types of reactions to the ball can be classified in four categories: no reaction, braking, avoidance and a combination of the latter two reactions. All these reactions occurred with a minimum change of 70% in the participants’ driving behavior when faced with a hazard, regardless of the vehicle (real or simulated), which amounts to a first degree level of concordance. These reactions presumably varied depending on to each individual’s ability to react and on their choice of what seems to be the best option for avoiding the hazard. For example, Venkatraman, Lee, & Schwarz [11] observed in a real-life situation that the distance to the obstacle influenced drivers’ patterns of deceleration and the choice of whether to deviate or not. This shows one of the advantages of establishing stable, reproducible protocols. With a sufficiently large sample and stable experimental situations, it is possible to compare participants’ driving and physiological behavior in simple driving situations *vs*. those involving a hazard. The results obtained here show that on the stretch of road where the event took place, the drivers’ average speed was lower (-6 kph) in the real vehicle than in the simulator, whereas the pattern of deceleration was identical. This means that a similar braking reaction to the ball occurred in both cases, as observed by Godley, Triggs, & Fildes [6], probably followed by an acceleration in order to return as quickly as possible to the initially imposed speed. As previously reported by Santos, Merat, Mouta, Brookhuis, & de Waard [57], greater speed variability occurs in a simulator than in a real car. As regards the present drivers’ responses to the stressful event, they braked strongly in front of the ball, both in the real vehicle and in the simulator. This reaction was previously observed in the case of rear-end collisions on a road [58], on a track [59] and in a simulator [8,60,61]. In addition to the longitudinal variability, the deviation of the vehicle induced by increasing the mean steering wheel angle was greater in the real car driving condition (+1.5°). Reactions of this kind to a hazard have previously been observed in simulated dynamic environments [1,8] and real environments [1,62], whereas the braking behavior observed in a static simulator was judged to be unrealistic [34]. In short, it can be said in the first place that the type of vehicle did not affect the drivers’ braking maneuvers in response to an unexpected obstacle. Secondly, the overall speed parameters and the value of the lateral deviation recorded at the arrival of the ball were lower under simulated conditions than on the track.

As regards the physiological parameters, an increase in heart rate, breathing rate and EDA phasic response can be expected to occur in response to a hazardous event [16,63]. This was what occurred in our study in the real vehicle: the heart rates increased by 4 bpm during a few tens of seconds after the event as previously described by Johnson et al. [25], the breath rates increased by 2 bpm and the phasic EDA increased two-fold, in line with previous findings by Schneegass et al. [18]. No changes in the heart rate were observed, however, in the simulator, which is not in agreement with previous simulator studies [20,21,64]. It is possible that simulator sickness symptoms may hide the classical physiological reactions induced by a stressor. However, it is worth noting that the Nausea subscale in the SSQ, which includes several items on body sensations, was above normal not only in the simulator but also under real life driving conditions. A second possibility is that the simulator settings in our experiment may not have been stressful enough to induce a change in the drivers’ physiological parameters: the ball may not have been perceived by the drivers as a threat because they were aware that the situation was virtual, involving no serious consequences to themselves. These physiological findings suggest a limit to the perceptions induced by the Sherpa2 simulator in drivers faced with a situation that is supposed to induce stress. The driving parameters are subject to a scale factor which makes participants’ simulated driving behavior only relatively valid since no physiological stress symptoms were induced in the simulator. This shows that it can be useful to analyze drivers’ feelings of presence via more than just one aspect, which may not always be a valid index. The significance of the LF/HF ratio, which showed no noteworthy inter-vehicle differences, will now be discussed below.

### Reproducibility and evolution of drivers’ behavior

One of the main advantages of using driving simulators is that simulated driving situations are highly reproducible. After the initial adaptation process, they can therefore be expected to induce reproducible driving behavior and physiological responses. In order to test this hypothesis, the same hazard was presented twice in this study to establish whether the repetition of drivers’ exposure to a hazard induced the same behavior with time. All the physiological parameters of interest increased in the first real life trial, and this increase was again observed in the second trial; whereas no physiological reactions were observed under simulated conditions in either group (G1S or G2S). Upon being exposed to a ball crossing the road in front of their vehicle, participants may have experienced some ’fear’ in the real car, as this event really involved a risk. This feeling of fear was not as strongly experienced in the simulator as the participants were running no physical risks in this set-up. This feeling of being invulnerable may considerably decrease the stress responses recorded in the simulator, in terms of the physiological parameters affected by the sympatho-vagal balance [13]. Nonetheless, the LF/HF ratio showed a similar pattern of evolution under both real and simulated conditions. In comparison with the first trial with no ball, the LF/HF ratio increased sharply on the first appearance of the ball and remained high in the subsequent trials (even without any ball crossing the road). It is usually assumed that the higher the LF/HF ratio, the more stressed individuals will be [13]. The term stress is frequently used instead of arousal [65]. Although the participants probably experienced stress during their first exposure to the ball (even if the simulated hazard was not strong enough to activate the sympathetic system) may have been triggered in both groups (as indicated by the LF/HF ratio). In the simulated condition, the sympathetic system may have been activated by the first hazardous event, enhancing the drivers’ level of arousal without eliciting a stress reaction in the ’fearful’ sense of the term. By contrast, in a real vehicle on the track, each hazardous event increased the sympathetic activity, resulting not only in a higher level of arousal but also in a strong stress reaction of the organism. All in all, these findings may not only explain the physiological variations measured but also the evolution of the participants’ driving reactions. An increase in the level of arousal would lead to an increase in attention [66] and a faster and stronger braking response in the second ball test (reflected in stronger deceleration levels). Participants had less need to combine braking with a steering wheel stroke to avoid the obstacle crossing the road. This behavior was observed in both groups G1R and G2S, whose number of steering wheel actions decreased with time, whereas greater deceleration was observed in these groups in trial 2 than in trial 1.

### Limitations

Real-world driving tests are quite difficult to set up in experimental settings. Due to methodological constraints, group G1 performed the real driving task first, followed by the simulated task. The second group of participants recruited began with the simulated task in order confirm that the results obtained with the first group were not induced by an order effect, and with a view to making comparisons between conditions. The statistical analysis of G1S and G2S showed the existence of no significant differences between the two groups. In addition, the most significant differences observed between G1R and G1S were also observed between G1R and G2S, which supports the idea that drivers’ behavior in the simulator was reproducible. In addition, as shown by the MEC-SPQ, it was questionable whether the situation on the track was true to life: in comparison with common driving situations, a track is a real but not a realistic environment. This brings us back to the ethical and safety limits encountered in research and development processes, which are constantly being overcome thanks to technological advances, but are still subject to some inevitable and difficult to overcome barriers.

### Conclusion

In the race for improving car safety, simulators are widely used tools. However, their validity is not yet absolute and needs to be further investigated. Validity is intrinsically linked to human factors because the driver in the car plays an important role which cannot be neglected. This role can be measured based on the concept of presence, which can be assessed using declarative questionnaires and measuring drivers’ behavior and physiological parameters, which to the best of our knowledge, have rarely been recorded all together in the same experiment. It was therefore proposed in this study to focus on presence on these three levels not only in a speed maintenance situation, but also at the occurrence of a hazardous event, i.e. a ball one meter in diameter crossing the road like a pedestrian rushing out in front of the vehicle. In the control situation (a speed maintenance test), the results confirmed the absolute or relative validity (with a scaling factor) of the Sherpa2 motion based driving simulator in comparison with a real vehicle as far as the driving behavior and physiological parameters were concerned. However, the stressful situation affected the drivers’ physiological parameters only in the real driving situation. Simulator sickness symptoms are still an issue, and the drivers’ declared feeling of presence were impacted by their belief in the environment through which they were travelling.

### Future work

These results have practical applications while at the same time questioning the validity of virtual reality, especially in the R&D setting. Further studies on these lines are now required in order to assess the validity of simulators, taking the concept of presence into account from various points of view, including behavioral, subjective and physiological parameters. It would be conceivable to equip the simulators with physiological sensors in a systematic way in order to get the most out of the possible observations. Thus, it would be easier and therefore more frequent in future studies to learn more about the relationships between ethological, psychological, physiological and emotional validity and to improve the settings of the simulators in a more thorough way. While focusing mainly on safety solutions, including driver monitoring systems, it is necessary to take into account the fact that drivers tend to show no physiological reactions to simulated hazards when car manufacturers carry out tests without the drivers being actually physically endangered. Despite their lack of controllability, the large set of data directly extracted from the vehicles on the road could usefully complement future studies by comparing the data collected in the simulator with data not only from real vehicles but also from real road hazard situations.
